# Mass spectrometric detection of biomarkers for early assessment of intraamniotic fluid infection

**DOI:** 10.1016/j.dib.2015.11.020

**Published:** 2015-11-20

**Authors:** Consuelo Cháfer-Pericás, Vedran Stefanovic, Ángel Sánchez-Illana, Javier Escobar, María Cernada, Elena Cubells, Antonio Núñez-Ramiro, Sture Andersson, Máximo Vento, Julia Kuligowski

**Affiliations:** a Neonatal Research Unit, Health Research Institute La Fe, Valencia, Avenida Fernando Abril Martorell 106, 46026 Valencia, Spain; b Department of Obstetrics and Gynecology, Fetomaternal Medical Center, Helsinki University Central Hospital and University of Helsinki, Haartmaninkatu 2, 00029 Helsinki, Finland; c Children׳s Hospital, University of Helsinki and Helsinki University Hospital, Stenbäckinkatu 11, PO Box 281, 00029 Helsinki, Finland; d Division of Neonatology, University & Polytechnic Hospital La Fe, Avenida Fernando Abril Martorell 106, 46026 Valencia, Spain

**Keywords:** Glutathione sulfonamide (GSA), Biomarker detection, Intraamniotic fluid infection, Time-of-flight mass spectrometry (TOF-MS), High performance liquid chromatography (HPLC), Liquid-chromatography coupled to tandem mass spectrometry (LC-MS/MS)

## Abstract

This data article contains information on glutathione sulfonamide (GSA) structural confirmation and purity after synthesis, as well as mass spectrometry acquisition parameters for the determination of GSA and other biomarkers for the early assessment of intraamniotic fluid infection in amniotic fluid samples (Cháfer-Pericás et al., 2015) [1]. GSA standards were synthesized and structural confirmation was carried out employing time-of-flight mass spectrometry (TOF-MS); purity was assessed by high performance liquid chromatography (HPLC) with UV detection. For optimization of the acquisition parameters of GSA and other biomarkers, individual analytical standard solution at a concentration of 1 µmol L^−^^1^ was injected into an Acquity – Xevo TQ liquid-chromatography coupled to tandem mass spectrometry (LC-MS/MS) system from Waters (Milford, MA, USA) operating in the positive electrospray (ESI^+^) mode. Mass spectrometric detection of 3-nitro-tyrosine (3NO2-Tyr), 3-chloro-tyrosine (3Cl-Tyr), 8-hydroxy-2′-deoxyguanosine (8OHdG), GSA and oxidized glutathione (GSSG) was carried out by multiple reaction monitoring (MRM). Linear response curves were calculated for each analyte normalizing the signal with peak areas of internal standards.

**Specifications Table**

**Table:** 

Subject area	*Medicine*
More specific subject area	*Obstetrics & Gynecology*
Type of data	*Figures & table*
How data was acquired	*TOF-MS, HPLC-UV, LC-ESI(+)-MS/MS*
Data format	*Structural confirmation/purity & LC-MS/MS acquisition parameters*
Experimental factors	*None applied*
Experimental features	*TOF-MS for structural elucidation and HPLC-UV for purity assessment of synthetized GSA; LC-ESI(+)-MS/MS acquisition parameters for each biomarker*
Data source location	*Valencia, Spain*
Data accessibility	*Data are with this article*

## 1 Value of the data

• GSA standard was synthetized and its molecular structure was confirmed by TOF-MS.
• Purity of the GSA standard was assessed employing HPLC-UV.
• LC-MS/MS for the simultaneous quantification of potential biomarkers in small sample volumes.
• MRM acquisition enabling a highly specific and sensitive analytical response.
• Linear response curves for each biomarker normalized by peak area values obtained from internal standard solutions for improved reproducibility.

## 2 Data, materials and methods

### 2.1 GSA standards

**Fig. 1:**
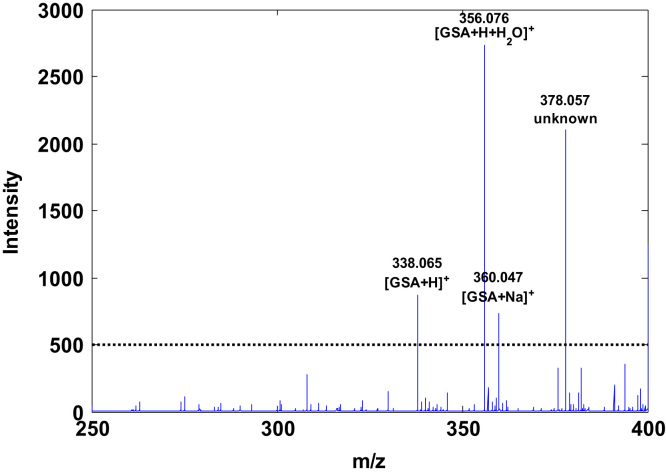
TOF-MS spectrum of GSA fraction. Note: Molecular formula of GSA: C10H15N3O8S, theoretical molecular weight of GSA: 337.05744.

**Fig. 2:**
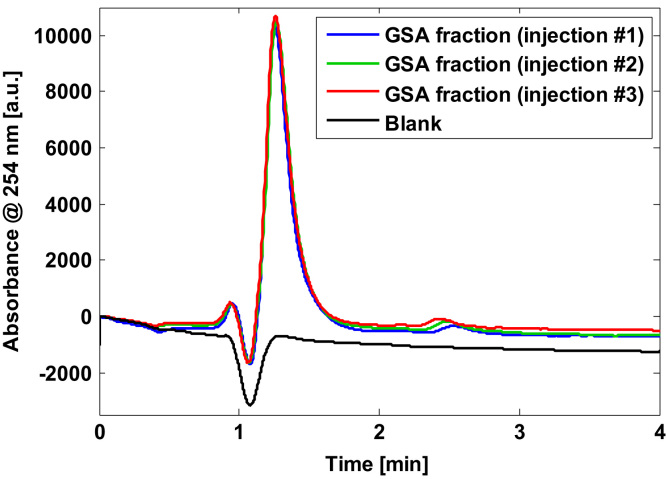
Chromatograms from a GSA containing fraction (triplicate injection) after purification and a blank injection.

GSA was synthesized and purified as described by Harwood et al. [2] by treating an aqueous GSH solution with hypochlorite solution in phosphate buffer (pH 7.4) at an equimolar ratio (100 mmol L^−^^1^) at room temperature. GSA was purified by manual collection of fractions from a Shimadzu Scientific Instruments (Columbia, MD, USA) high performance liquid chromatographic (HPLC) system equipped with a Halo C18 column (2.1×100 mm, 2.7 µm) from Advanced Materials Technology (Wilmington, DE, USA) and using an injection volume of 50 µL. Chromatograms were acquired using UV detection at 222 and 254 nm. Separation was achieved using an H_2_O (0.1% formic acid): methanol gradient and a flow rate of 0.5 mL min^−1^; gradient conditions were set as follows: from 0 to 3 min 2% methanol; from 3 to 6 min from 2% to 98% methanol; from 6 to 10 min 98% methanol; from 10 to 10.1 min from 98% to 2% methanol and from 10.1 to 14 min 2% methanol. The collected fractions were lyophilized to obtain a fluffy white powder which at −20 °C was stable over a period of several months. In the obtained fractions, the presence of GSA was confirmed by recording full scan direct infusion TOF-MS spectra employing an ABSciex 5600-Triple TOF-MS spectrometer (Framingham, MA, USA) operating in the ESI^+^ mode. Fig. 1 shows a TOF-MS spectrum of a GSA containing fraction in the region between 250 and 400 m/z. Four peaks with intensities >500 counts were identified and three of them could be assigned to the molecular ion of GSA and GSA adducts, respectively. The purity of the obtained GSA was determined from three independent runs to be 91±2% employing the above-described HPLC-UV method and measuring the GSA peak area vs. the total area under the curve in chromatograms recorded at 254 nm. Fig. 2 shows chromatograms of a GSA containing fraction and a blank injection.

### 2.2 LC-MS/MS acquisition parameters for biomarker determination in AF samples

An Acquity – Xevo TQ from Waters (Milford, MA, USA) operating in the ESI^+^ mode was used for the analysis of standard solutions and processed amniotic fluid samples (details can be found in [1]). Parameters were set as follows: capillary voltage was 3.2 kV, source temperature was 150 °C, desolvation temperature was 395 °C, nitrogen cone and desolvation gas flows were 150 and 800 L h^−1^, respectively and the dwell time was 5 ms. For MS parameter optimization, individual standard solutions of each biomarker at a concentration of 1 µmol L^−^^1^ were injected. For LC separations, a Kinetex C8 column (100×2.1 mm, 1.7 µm) from Phenomenex was used running a gradient employing H_2_O (0.1% v/v formic acid) and acetonitrile (0.1% v/v formic acid) as mobile phases A and B with gradient conditions as follows: from 0 to 1.25 min 1% B; from 1.25 to 4.75 min the mobile phase composition changed from 1% to 98% B; from 4.75 to 5 min 98% B; from 5 to 5.1 min the gradient returned to its initial conditions of 1% B which were held until 6 min. Flow rate, column temperature and injection volume were set to 0.4 mL min^−^^1^, 45 °C and 2 µL, respectively. Samples were kept in the autosampler at 4 °C during batch analysis. Multiple reaction monitoring (MRM) was carried out employing the acquisition parameters summarized in Table 1. MassLynx 4.1 and QuanLynx 4.1 from Waters (Milford, MA, USA) were used for data acquisition and processing, respectively. Linear response curves were calculated for each biomarker normalizing with peak areas of internal standards.

## Conflict of interest

The authors declare that there is no conflict of interest on any work published in this paper.

## Figures and Tables

**Table 1:** LC-ESI(+)-MS/MS acquisition parameters.

**Analyte**	***m*/*z* parent ion**	**Cone [V]**	***m*/*z* daughter ion**	**CE [eV]**
3NO_2_-Tyr	227.1	25	181	10
3Cl-Tyr	216	30	170	15
8OHdG	284	30	168	15
GSA	338	45	155.1	25
GSSG	613.2	50	355	35
8OHdG-C13N15	284	30	168	15
Phe-D5	171.5	30	125	20
Meth-D3	153	30	63.5	25

CE: collision energy; 3NO_2_-Tyr: 3-nitro-tyrosine; 3Cl-Tyr: 3-chloro-tyrosine; 8OHdG: 8-hydroxy-2׳ -deoxyguanosine; GSA: glutathione sulfonamide; GSSG: oxidized glutathione; 8OHdG-C13N15: 8-hydroxy-2׳-deoxyguanosine-C13,N15; Phe-D5: phenylalanine-D5, Meth-D3: methionine-D3*.*
